# Comparative study on the efficacy of conservative treatment and surgical treatment for simple Mason II radial head fracture

**DOI:** 10.3389/fsurg.2026.1842557

**Published:** 2026-06-23

**Authors:** Wei Fan, Na Yang, Rui Qiao, Chen Wang, Chen Fei, Kun Zhang, Zhe Song

**Affiliations:** Honghui Hospital, Xi’an Jiaotong University, Xi’an, China

**Keywords:** conservative treatment, internal fixation, Mason classification, open reduction, radial head fracture

## Abstract

This study aimed to analyze the therapeutic effects of conservative fixation with braces vs. open reduction with internal fixation surgery for simple Mason II radial head fractures.

**Methods:**

Data from 65 patients with Mason type II radial head fractures treated at the Orthopedics Department of Xi'an Honghui Hospital between January 2018 and January 2021 were analyzed retrospectively. The patients’ age range was 18–65 years (mean age, 37.7 years). Thirty-six patients underwent surgical treatment, and 29 underwent conservative treatment. Fracture healing time, elbow joint range of motion, forearm rotation block, pain VAS score, and Broberg–Morrey elbow joint function score were recorded and compared.

**Results:**

All 65 patients completed follow-up. The conservative-treatment group was followed for 3–12 months (mean 5.14 ± 2.13 months). Fracture healing time was 10.41 ± 1.31 weeks. Mean elbow flexion was 134.32° ± 8.59°, extension 7.65° ± 8.27°, and Broberg–Morrey score 90.82 ± 15.51. One patient experienced fracture malunion. The surgical-treatment group was followed for 12–36 months (mean 17.94 ± 4.90 months). Fracture healing time was 10.92 ± 2.78 weeks. Mean elbow flexion was 129.27° ± 15.72°, extension 5.26° ± 5.98°, and Broberg–Morrey score 91.44 ± 17.12. There were two cases of postoperative infection and two cases of internal-fixation failure. No significant intergroup differences were found in fracture healing time, elbow range of motion, forearm rotation, Broberg–Morrey score, or complication rate (*P* > 0.05). Subgroup analysis showed no significant differences related to gender or hand dominance. In the surgical group, screw fixation and mini plate fixation yielded similar outcomes.

**Conclusion:**

Both conservative and surgical treatments achieve favorable bone healing and satisfactory elbow function in simple Mason II fractures. Preoperative CT improves fracture evaluation. Treatment should be individualized based on comprehensive assessment.

## 1 Introduction

Radial head fracture is one of the most common elbow joint fractures and accounts for 2%–6% of systemic fractures and 17%–44% of elbow joint fractures (1–4). According to the Mason classification, type II radial head fractures involve displacement >2–3 mm and involvement of >30% of the radial head articular surface. The optimal treatment for this fracture type remains controversial. Shukla et al. (5). suggested that radial head depression of 2 mm or angulation of 30° may cause up to 80% loss of concave compressive stability of the radiocapitellar joint, thereby compromising elbow stability. Long-term articular incongruity also raises concerns regarding the subsequent development of post-traumatic elbow osteoarthritis (6).Some authors advocate surgical treatment using mini plates, headless screws, or elastic intramedullary nails (7, 8). Others support conservative treatment, which avoids surgical complications such as elbow pain, stiffness, and forearm weakness (9, 10). This study compared the clinical efficacy of conservative and surgical treatment for simple Mason II radial head fractures to provide evidence-based treatment options.

## 2 Materials and methods

### 2.1 General information

A total of 65 patients (35 males, 30 females; aged 18–65 years) with simple Mason II radial head fracture were retrospectively analyzed. Patients were divided into conservative-treatment group (*n* = 29; 15 males, 14 females) and surgical-treatment group (*n* = 36; 20 males, 16 females). All patients had isolated radial head fractures without combined injuries.

### 2.2 Inclusion and exclusion criteria

**Inclusion criteria**:

1. Fresh simple Mason II radial head fracture;
2. Age 18–65 years;
3. Clear history of trauma;
4. Preoperative CT and elbow x-rays available.

**Exclusion criteria**:

1. Old fracture, pathological fracture, or open fracture;
2. Patients with obvious elbow ligament injury accompanied by elbow instability;
3. Concomitant elbow or forearm injuries;
4. Inability to complete follow-up or functional exercise.

### 3.3 Therapeutic method

#### 3.3.1 Conservative-treatment group

For the conservative-treatment group, the feasibility of conservative treatment was explained to the patients during treatment, emphasizing the importance of elbow functional exercise. After the patient visited the hospital, an adjustable brace for the elbow joint was given for elbow flexion of 90°, and the forearm was fixed in a neutral position. After 24 h of fixation, finger and wrist flexion and extension activities were started, while elbow flexion and extension and forearm rotation activities were temporarily stopped. One week after the injury, radiography of the forearm was conducted at the outpatient department for follow-up. After the fracture position was confirmed to be normal, active elbow flexion and extension and forearm rotation activities were started, 2–3 times a day, at least 5 min each time. During the rest of the time, the elbow brace was fixed continuously to reduce the probability of fracture displacement. The patient was informed to gradually increase the range of activities and adopt the progressive exercise mode. All of the patients were followed up weekly within 1 month after injury, monthly from 1 month to 3 months after injury, at 6 months after injury, and once every 6 months thereafter. The patients were encouraged to actively carry out functional exercise, and try to ensure that elbow joint activities return to near normal within 4 weeks after injury.

#### 3.3.2 Surgical-treatment group

**Figure 1:**
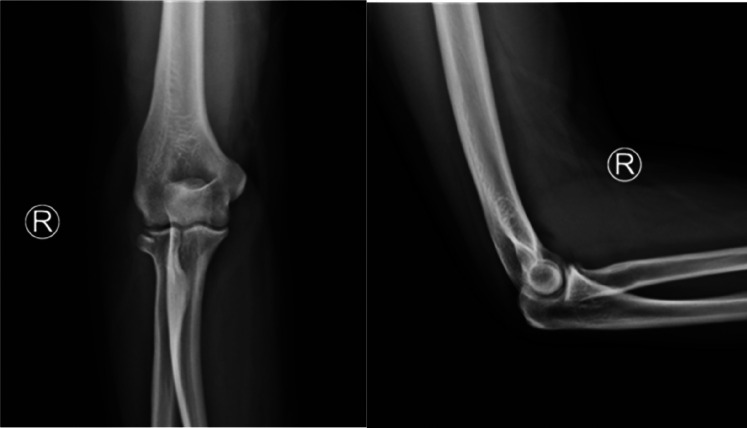
Zhang XX, male. The patient suffered from pain and limited motion of the right elbow joint after a fall. Diagnosis: Mason type II radial head fracture of the right side. Surgical method: Open reduction and screw internal fixation. Preoperative AP and lateral radiographs demonstrated significant fracture displacement.

**Figure 2:**
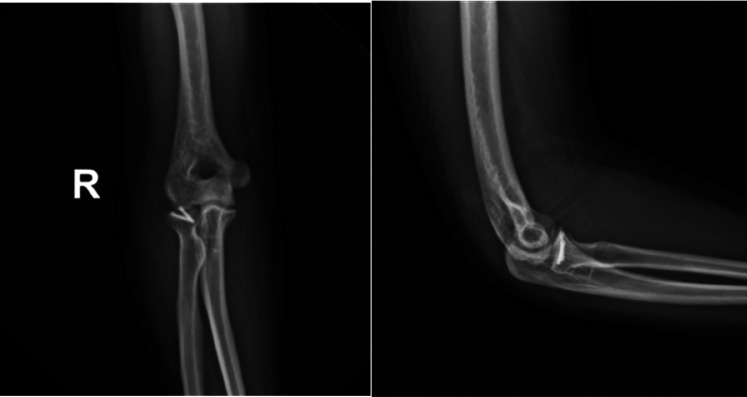
Zhang XX, male. The patient suffered from pain and limited motion of the right elbow joint after a fall. Diagnosis: Mason type II radial head fracture of the right side. Surgical method: Open reduction and screw internal fixation. 1-month postoperative AP and lateral radiographs, fixed with 2 headless compression screws.

**Figure 3:**
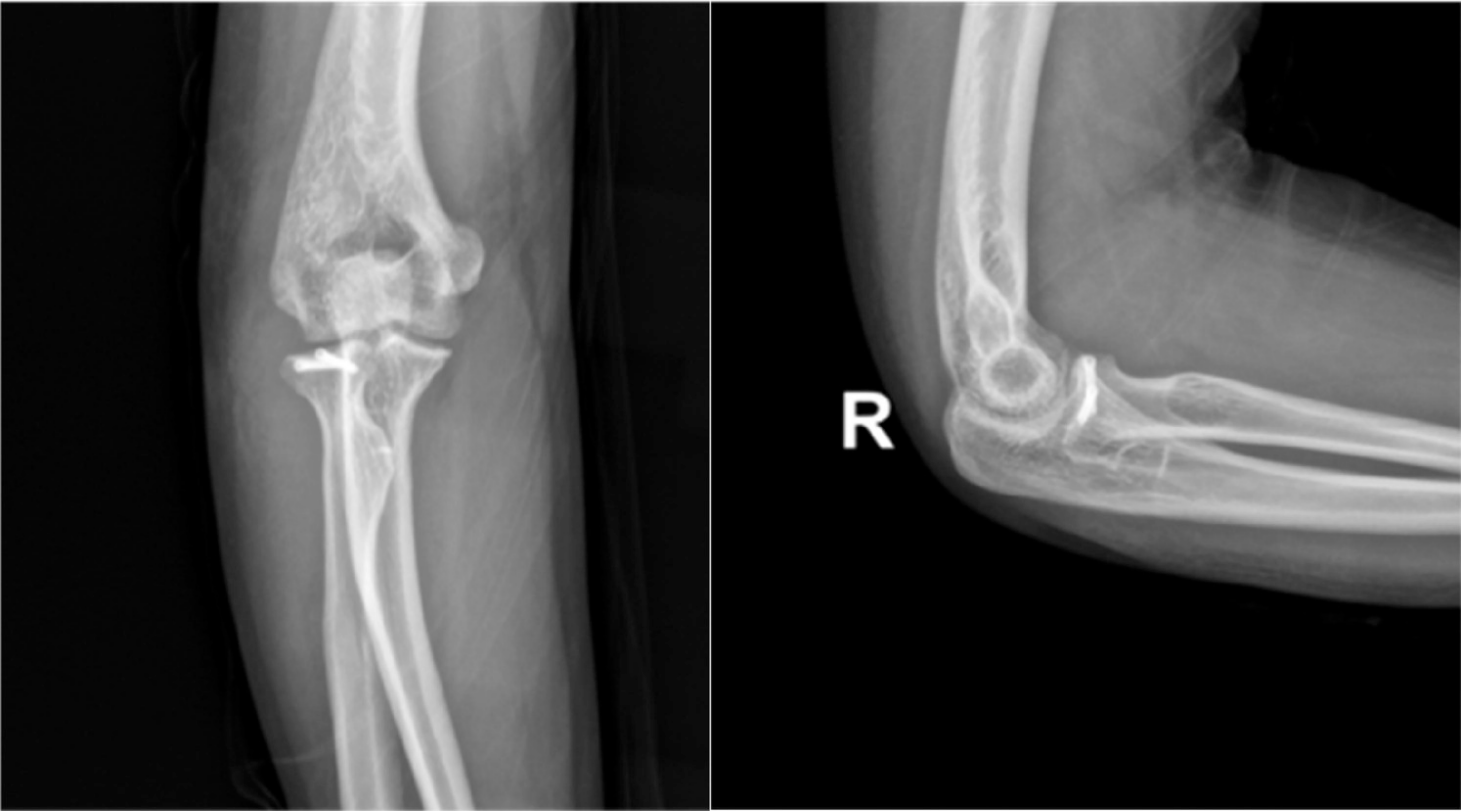
Zhang XX, male. The patient suffered from pain and limited motion of the right elbow joint after a fall. Diagnosis: Mason type II radial head fracture of the right side. Surgical method: Open reduction and screw internal fixation. 3-month postoperative AP and lateral radiographs, fracture union achieved.

**Figure 4:**
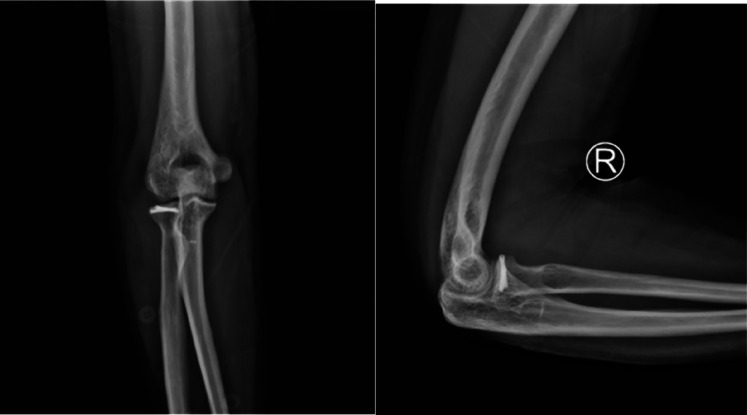
Zhang XX, male. The patient suffered from pain and limited motion of the right elbow joint after a fall. Diagnosis: Mason type II radial head fracture of the right side. Surgical method: Open reduction and screw internal fixation. 5-month postoperative AP and lateral radiographs, fracture union achieved.

**Figure 5:**
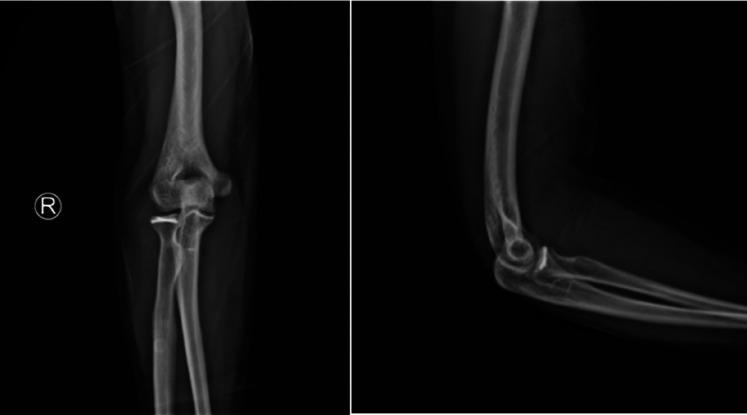
Zhang XX, male. The patient suffered from pain and limited motion of the right elbow joint after a fall. Diagnosis: Mason type II radial head fracture of the right side. Surgical method: Open reduction and screw internal fixation. 13-month postoperative AP and lateral radiographs, fracture union, satisfactory internal fixation position, and no heterotopic ossification.

**Figure 6:**
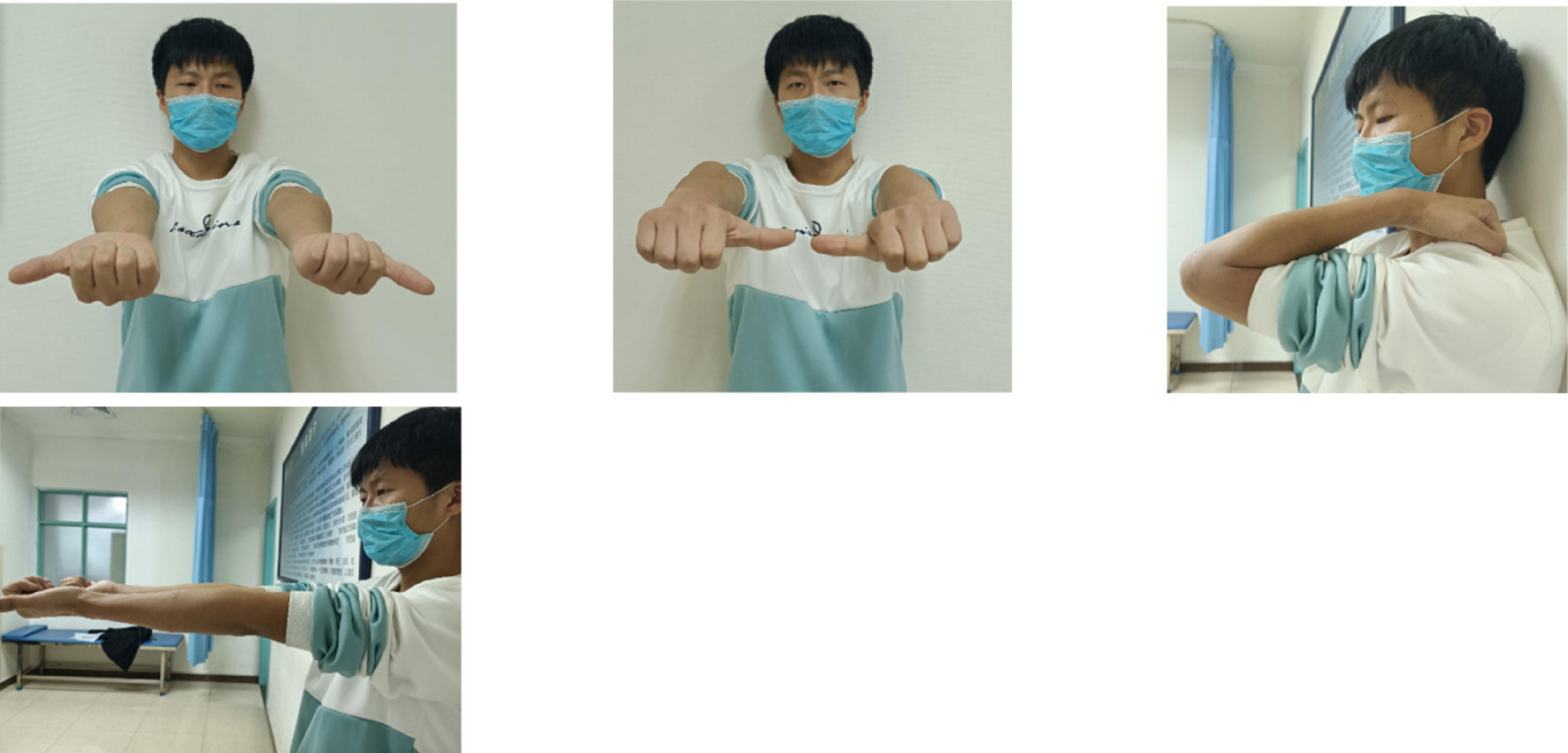
Zhang XX, male. The patient suffered from pain and limited motion of the right elbow joint after a fall. Diagnosis: Mason type II radial head fracture of the right side. Surgical method: Open reduction and screw internal fixation. Elbow functional recovery at 13 months after surgery:Excellent elbow functional recovery.

For the surgical-treatment group, all operations were performed by deputy chief physicians with equivalent qualifications and experience. The patients were placed in a supine position and anesthetized by nerve block or general anesthesia. The upper air bag tourniquet adopted the Kocher approach on the lateral side of the elbow joint, entered between the extensor carpi ulnaris and the elbow muscle, incised the annular ligament, exposed the radial head fracture, carefully cleared the congestion and clots around the joint and the broken end of the fracture, reduced the fracture, used countersunk screws or micro anatomical locking bone plate to fix the fracture, used countersunk screws for countersunk treatment, and placed the bone plate in the safe area. Intraoperative fluoroscopy determined the fracture reduction and internal fixation position, and confirmed that the fracture reduction was satisfactory. After the internal fixation position was good, the wound was rinsed, sutured, and closed layer by layer. The active and passive flexion and extension activities of the elbow joint started on the third day after operation, and the range of activities gradually increased. The patients were followed up every month from January to March after operation, and every 6 months after operation. They were also encouraged to actively carry out functional exercise, and strive to restore elbow joint activity to near normal within 4 weeks after operation. Typical surgical cases are shown in Figures 1–6.

### 2.4 Evaluation index

All patients were followed up at the outpatient department with anteroposterior and lateral elbow radiographs. Fracture healing time was recorded. At initial presentation, elbow range of motion and forearm rotation block were evaluated. At the final follow-up, elbow flexion-extension, forearm rotation, and Broberg–Morrey elbow function score were assessed.

Broberg–Morrey score (total 100 points)

Pain: 45 points
Range of motion: 20 points
Joint stability: 10 points
Daily function: 25 points

**Grading criteria**:

Excellent: 90–100
Good: 80–89
Fair: 70–79
Poor: <70

### 2.5 Statistical analysis

Data were analyzed using SPSS 19.0. Continuous data are presented as mean ± standard deviation and compared using independent-samples *t*-test. Categorical variables were compared using chi-square or Fisher exact test. Subgroup analyses were performed according to gender, hand dominance, and surgical fixation method. *P* < 0.05 was considered statistically significant.

## 3 Results

### 3.1 Conservative-treatment group

Twenty-nine patients were followed for 3–12 months (mean 5.14 ± 2.13 months). Fracture healing time was 8–12 weeks (mean 10.41 ± 1.31 weeks). Mean elbow flexion was 134.32° ± 8.59°, extension 7.65° ± 8.27°. Mean Broberg–Morrey score was 90.82 ± 15.51. One patient developed malunion of the radial head fracture with severe impairment of elbow joint function. Radial head replacement combined with elbow joint release was performed at 6 months after injury, and the patient achieved satisfactory functional recovery postoperatively.

### 3.2 Surgical-treatment group

Thirty-six patients were followed for 12–36 months (mean 17.94 ± 4.90 months). Fracture healing time was 8–16 weeks (mean 10.92 ± 2.78 weeks). Mean elbow flexion was 129.27° ± 15.72°, extension 5.26° ± 5.98°. Mean Broberg–Morrey score was 91.44 ± 17.12. A total of four postoperative complications were observed in this cohort, including two cases of surgical site infection that resolved completely after repeated wound debridement and dressing changes. The remaining two patients experienced internal fixation failure; however, both fractures achieved bony union with acceptable elbow functional recovery, and subsequent implant removal was performed in the late follow-up period.

### 3.3 Initial range of motion and forearm rotation block

At presentation, 11 patients in the conservative group and 14 patients in the surgical group had limited forearm rotation. Final range of motion and functional scores were not significantly affected by preoperative rotation block.

### 3.4 Subgroup analysis by gender and hand dominance

In both groups, no significant differences were found in fracture healing time, elbow motion, or Broberg–Morrey scores between males and females or between dominant and non-dominant arms (all *P* > 0.05).

### 3.5 Comparison of fixation methods in surgical group

Thirty-six patients were treated with cannulated screws (*n* = 20) or mini plates (*n* = 16). No significant differences were observed between the two subgroups in healing time, elbow motion, functional score, or complication rate (*P* > 0.05) (Tables 1–3).

**Table 1:** Comparison of elbow range of motion.

Group	*n*	Flexion	Extension
Conservative-treatmen*t* group	29	134.32° ± 8.59°	7.65° ± 8.27°
Surgical-treatment group	36	129.27° ± 15.72°	5.26° ± 5.98°
*P*	—	*P* = 0.920[−8.414,9.307]	*P* = 0.406[−2.009,4.907]

**Table 2:** Broberg–Morrey score and fracture healing time.

Group	*N*	Broberg–Morrey score	Fracture healing time
Conservative-treatment group	29	90.82 ± 15.51	10.41 ± 1.31
Surgical-treatment group	36	91.44 ± 17.12	10.92 ± 2.78
P	—	*P* = 0.655[−5.623,8.880]	*P* = 0.508[−0.916,1.831]

**Table 3:** Postoperative complications.

Complications	Group	*n*	Impact on elbow joint function	Treatment methods
Infection	Surgical-treatment group	2	Minimal	Wound debridement and dressing changes
Internal fixation failure	Surgical-treatment group	2	Minimal	Implant removal after fracture healing
Developed malunion	Conservative-treatment group	1	Severe	Radial head replacement

### 3.6 Intergroup comparisons

There were no significant differences between the conservative and surgical groups in fracture healing time, elbow flexion and extension, forearm rotation, or Broberg–Morrey score (all *P* > 0.05).

## 4 Discussion

As an important bone structure for the stability of the elbow joint, the radial head is the second important bone structure besides the olecranon of the ulna. When the radial head is fractured, its anatomical structure should be restored as much as possible to maintain the stability of the elbow joint. The radial head is disc-shaped. The disc-shaped articular surface and the capitulum of the humerus form the humeroradial joint. The annular articular surface of the radial head and the ulnar radial notch form the upper ulnoradial joint. Except for the outer 110°, the other articular surfaces enter the radial notch when rotating. Heim summarized the static stability system of the elbow joint as the four-column theory of the elbow joint stability ring, namely the medial column, the lateral column, the anterior column, and the posterior column, in which the radial head, the capitulum humeri, and the lateral collateral ligament form the lateral column. The radial head primarily maintains the lateral stability and stress conduction of the elbow joint. About 60% of the stress load of the forearm is conducted by the radial head. At the same time, the radial head provides 20%–30% of the valgus stability of the elbow joint. When subjected to valgus stress, it can reduce the tension of the medial aspect of the elbow joint and combat the valgus rotation instability of the elbow joint. Therefore, when the radial head fracture occurs, it should be treated correctly as soon as possible to ensure that its impact on elbow stability and even elbow function is reduced as much as possible.

There are many types of radial head fracture, and the most common classification used in clinical practice is Mason classification, which has guiding significance for clinical treatment. Mason classified the radial head fracture into four types according to the size of the fracture block, the degree of displacement, and whether it is accompanied by combined injury as follows: type I, fracture of the radial head or neck without displacement (or displacement <2 mm); type II, fracture displacement more than 2–3 mm, and the fracture involves more than 30% of the radial head; type III, comminuted fracture; and type IV, elbow dislocation in any of the above types. There is a consensus in the academic community on the treatment of type I radial head fracture; namely, conservative treatment is considered capable of achieving ideal therapeutic effect (11). As type III and type IV radial head fractures are comminuted fractures or fractures with elbow dislocation, usually surgical treatment can restore the anatomical structure of the radial head and satisfactory elbow function.

There has been a great controversy about the choice of treatment for Mason type II fractures, especially for simple radial head fractures. Some scholars believe that simple type II radial head fracture can be treated conservatively. Domestic scholars Zha et al. (12) and Liang et al. (13) have specifically studied the conservative treatment of Mason type II fractures. They believe that after conservative treatment of Mason type II fractures, elbow function can be restored close to normal, without complications such as fracture nonunion, ischemic necrosis, and joint degeneration. Foreign scholars Akesson et al. (9) and Yoon et al. (14) found that when the fracture displacement was 2–5 mm and the articular surface involvement was >30%, there was no significant difference in clinical function and elbow mobility between surgical treatment and conservative treatment, and patients with conservative treatment had no noticeable discomfort in the long-term follow-up. Even in athletes, conservative treatment can restore the pre-injury sports level (15). The main braking methods of conservative treatment are plaster fixation, brace fixation, sling, and elastic bandage fixation. Currently, the most commonly used are plaster and brace fixation, which can better fix the fracture. However, because long-term braking leads to elbow dysfunction, it is generally recommended that the fixation time should not exceed 3 weeks. Therefore, functional exercise as early as possible can restore elbow function to the greatest extent, and muscle contraction activities should be started after 24 h of fixation. Functional exercise at this time inevitably leads to limb pain. It is recommended that patients continue functional exercise after pain relief. It has been shown (16, 17) that guiding patients to correctly understand pain during conservative treatment of functional exercise can significantly increase the recovery of elbow range of motion.

Compared with conservative treatment, more scholars believe that Mason type II radial head fracture should be treated with open reduction and internal fixation as soon as possible (18–20), so as to restore the bone anatomy of the radial head to the greatest extent, and restore the stability of the elbow joint.Failure to restore anatomical reduction of intra-articular fractures may lead to secondary elbow osteoarthritis (21). Surgical intervention is necessary especially when bony impingement restricts forearm rotation. It was proposed that hollow countersunk screws, absorbable screws, mini plates, and other (22) methods can be used for internal fixation of fractures. Furthermore, firm internal fixation can provide solid fixation support for early functional exercise, reduce the risk of fracture displacement in early elbow functional exercise, and make patients’ elbow function recover to the greatest extent. After surgical treatment, it is recommended to carry out elbow function exercise as soon as possible. Active and passive elbow flexion and extension function training should be carried out under the protection of brace on the second day after operation. After removing the brace 3 weeks after operation, elbow rotation and resistance training should be actively carried out. However, surgical treatment also has its shortcomings, and the complications brought by surgery are unavoidable, such as postoperative wound infection, elbow pain, stiffness, forearm weakness, radial nerve injury, and other complications (10). At the same time, surgery significantly increases the economic burden of patients' treatment.

In this study, patients with simple Mason type II radial head fracture treated with conservative treatment and surgical treatment were compared in terms of fracture healing time, elbow flexion activity, extension activity, Broberg–Morrey score, and excellent and good rate. We found that the two treatment methods for simple Mason type II radial head fracture achieved good clinical efficacy, and no significant differences were found in the observation index of comparative analysis. This is consistent with the systematic review published by Fabian et al. (23) in 2021, who conducted a systematic analysis of 11 articles on the treatment of Mason type II radial head fracture published between 2011 and 2020. The total sample size of the cases was 319 cases, of which 218 cases were treated with surgery and 101 cases were treated with conservative treatment. The authors compared the two groups of patients by Broberg–Morrey score, and did not find differences between the two treatment methods. However, they also mentioned that the probability of subsequent secondary surgery was higher for surgical patients than for patients undergoing conservative treatment, but the probability of arthritis caused by radial head fracture in the conservative-treatment group was more likely.

Two cases of internal fixation failure occurred in the surgical group, both of which were managed with plate fixation. Plate fracture was identified during postoperative radiographic follow-up. Despite hardware failure, satisfactory bony union and acceptable elbow functional recovery were achieved in both patients, and subsequent implant removal was performed. The exact mechanism of plate breakage remained undetermined. According to patient self-reports, elbow range of motion fully recovered without discomfort at six weeks postoperatively. The patients then resumed routine daily activities, household chores, and physical labor, after which elbow pain developed. Radiographs confirmed plate fracture, and brace immobilization was therefore administered. Complete fracture union was confirmed at the 16-week follow-up visit. Premature resumption of heavy physical activity and household work was presumed to be the primary cause of fixation failure. For cases complicated by postoperative bony nonunion, bone grafting and refixation represent viable therapeutic options (24, 25).In the conservative group, one patient presented with fracture malunion that severely compromised elbow function. The patient underwent radial head replacement combined with elbow release at six months after injury, with favorable postoperative functional outcomes. The relatively short mean follow-up period in the conservative group may have precluded the detection of late-onset elbow osteoarthritis secondary to residual articular surface irregularity, which might potentially influence the overall study results. However, as reported by Kachooei et al. (6) radiocapitellar arthritis occurs in merely 0.1% of patients following isolated radial head fractures. Such degenerative changes are more likely attributed to concomitant humeral articular injury rather than routine articular wear, suggesting that the risk of post-traumatic radiocapitellar arthritis is negligible in patients with simple radial head fractures.

In conclusion, the conservative treatment and surgical treatment of Mason type II radial head fracture can achieve good fracture healing and satisfactory elbow function, but the two treatment methods still have their own shortcomings. Therefore, for Mason type II radial head fracture, clinicians should comprehensively evaluate patients to determine personalized and precise treatment plan, so as to help patients recover as soon as possible.

## Data Availability

The original contributions presented in the study are included in the article/Supplementary Material, further inquiries can be directed to the corresponding author.

## References

[1] Duckworth AD Clement ND Jenkins PJ Aitken SA Court-Brown CM McQueen MM. The epidemiology of radial head and neck fractures. J Hand Surg Am. (2012) 37(1):112–9. 10.1016/j.jhsa.2011.09.034 22119600

[2] Pogliacomi F Schiavi P Pedrazzini A Nosenzo A Tocco S Ceccarelli F. Modified Mason type III and IV radial head fractures: results of different surgical treatments. Acta Biomed. (2015) 86(3):242–50. 26694151

[3] Sheehan SE Dyer GS Sodickson AD Patel KI Khurana B. Traumatic elbow injuries: what the orthopedic surgeon wants to know. Radiographics. (2013) 33(3):869–88. 10.1148/rg.333125176 23674780

[4] Shi Y Wang GF Mei K Zhang J Yun CJ Qian C. Clinical and radiographic outcomes of treatment of comminuted Mason type II radial head fractures with a new implant. Medicine (Baltimore). (2018) 97(13):e0086. 10.1097/MD.0000000000010086 29595630 PMC5895426

[5] Shukla DR Fitzsimmons JS An KN O’Driscoll SW. Effect of radial head malunion on radiocapitellar stability. J Shoulder Elbow Surg. (2012) 21(6):789–94. 10.1016/j.jse.2011.12.001 22521392

[6] Kachooei AR Ring D. Evaluation of radiocapitellar arthritis in patients with a second radiograph at least 2 years after nonoperative treatment of an isolated radial head fracture. Arch Bone Jt Surg. (2017) 5(6):375–9. 29299491 PMC5736885

[7] Li T Liu G Jiang X. Advances in treatment of fractures of the radius. Chin J Traumatol. (2017) 33(5):424–9. 10.3760/cma.j.issn.1001-8050.2017.05.008

[8] Jia Z Hong Y Li C Lin J Hu X. The clinical efficacy of the minimally invasive treatment of Mason type II radial head fractures using intramedullary fixation with double titanium elastic nails. Am J Transl Res. (2021) 13(11):12807–15. 34956495 PMC8661165

[9] Åkesson T Herbertsson P Josefsson PO Hasserius R Besjakov J Karlsson MK. Primary nonoperative treatment of moderately displaced two-part fractures of the radial head. J Bone Joint Surg Am. (2006) 88(9):1909–14. 10.2106/JBJS.E.01052 16951104

[10] Lindenhovius AL Felsch Q Ring D Kloen P. The long-term outcome of open reduction and internal fixation of stable displaced isolated partial articular fractures of the radial head. J Trauma. (2009) 67(1):143–6. 10.1097/TA.0b013e31818234d6 19590324

[11] Duckworth AD Wickramasinghe NR Clement ND Court-Brown CM McQueen MM. Long-term outcomes of isolated stable radial head fractures. J Bone Joint Surg Am. (2014) 96(20):1716–23. 10.2106/JBJS.M.01354 25320198

[12] Zhang Z Jiang X Gong M. Conservative treatment of the elbow osteonephrosis in Mason II and III fractures: efficacy. Chin J Orthop. (2018) 38(1):16–22. 10.3760/cma.j.issn.0253-2352.2018.01.003

[13] Liang J Xiang W Li J. Conservative treatment of stable radial osteoarthritis: a retrospective cohort study. Chin J Reconstr Surg. (2020) 28(4):369–71. 10.3977/j.issn.1005-8478.2020.04.17

[14] Yoon A King GJ Grewal R. Is ORIF superior to nonoperative treatment in isolated displaced partial articular fractures of the radial head? Clin Orthop Relat Res. (2014) 472(7):2105–12. 10.1007/s11999-014-3541-x 24577616 PMC4048435

[15] Guzzini M Vadalà A Agrò A Di Sanzo V Pironi D Redler A. Nonsurgical treatment of Mason type II radial head fractures in athletes. A retrospective study. G Chir. (2017) 37(5):200–5. 10.11138/gchir/2016.37.5.200 28098055 PMC5256901

[16] Egol KA Haglin JM Lott A Fisher N Konda SR. Minimally displaced, isolated radial head and neck fractures do not require formal physical therapy: results of a prospective randomized trial. J Bone Joint Surg Am. (2018) 100(8):648–55. 10.2106/JBJS.17.01023 29664851

[17] Teunis T Thornton ER Guitton TG Vranceanu AM Ring D. Coaching of patients with an isolated minimally displaced fracture of the radial head immediately increases range of motion. J Hand Ther. (2016) 29(3):314–9. 10.1016/j.jht.2016.02.003 27496986

[18] Tian X Dong J. Treatment strategies for elbow fractures: a review. Chin J Orthop. (2022) 42(4):251–7. 10.3760/cma.j.cn121113-20211017-00596

[19] Hackl M Wegmann K Hollinger B El-Zayat BF Seybold D Gühring T. Surgical revision of radial head fractures: a multicenter retrospective analysis of 466 cases. J Shoulder Elbow Surg. (2019) 28(8):1457–67. 10.1016/j.jse.2018.11.047 30713065

[20] Liu G Chen E Xu D Ma W Zhou L Chen J. Open reduction and internal fixation with bone grafts for comminuted mason type II radial head fractures. BMC Musculoskelet Disord. (2018) 19(1):288. 10.1186/s12891-018-2214-4 30111311 PMC6094563

[21] Rollo G Rotini R Eygendaal D Pichierri P Bisaccia M Prkic A. Effect of trochleocapitellar index on adult patient-reported outcomes after noncomminuted intra-articular distal humeral fractures. J Shoulder Elbow Surg. (2018) 27(7):1326–32. 10.1016/j.jse.2018.02.073 29907373

[22] Tarallo L Mugnai R Rocchi M Capra F Catani F. Comparison between absorbable pins and mini-screw fixations for the treatment of radial head fractures Mason type II–III. BMC Musculoskelet Disord. (2018) 19(1):94. 10.1186/s12891-018-2014-x 29587695 PMC5872384

[23] Lanzerath F Hackl M Wegmann K Müller LP Leschinger T. The treatment of isolated Mason type II radial head fractures: a systematic review. J Shoulder Elbow Surg. (2021) 30(3):487–94. 10.1016/j.jse.2020.10.011 33197586

[24] Rollo G Vicenti G Rotini R Prkic A Eygendaal D Meccariello L. Open reduction and internal fixation using double plating with biological and artificial bone grafting of aseptic non-unions of the distal humerus: clinical results. Strategies Trauma Limb Reconstr. (2021) 16(3):144–51. 10.5005/jp-journals-10080-1533 35111253 PMC8778730

[25] Rollo G Luceri F Bisaccia M Lanzetti RM Luceri A Agnoletto M. Allograft versus autograft in forearm aseptic non-union treatment. J Biol Regul Homeost Agents. (2020) 34(4 Suppl. 3):207–12. 33261279

